# Eye care service utilization and associated factors among older adults in Hawassa city, South Ethiopia

**DOI:** 10.1371/journal.pone.0231616

**Published:** 2020-04-16

**Authors:** Efa Derecha Morka, Betelhem Temesgen Yibekal, Mebratu Mulusew Tegegne

**Affiliations:** 1 Department of Ophthalmology and Optometry, School of Medicine, College of Medicine and Health Science, Hawassa University, Hawassa town, Ethiopia; 2 Department of Optometry, School of Medicine, College of Medicine and Health Science, University of Gondar, Gondar town, Ethiopia; University of Montreal, CANADA

## Abstract

**Purpose:**

The study aimed to assess the proportion of eye care service utilization and associated factors among older adults age ≥ 40 years in Hawassa city, South Ethiopia, June 2019.

**Method:**

A community-based cross-sectional study was conducted among adults aged 40 years and above who permanently live in Hawassa city from April 25 to May 30, 2019. Multistage sampling technique was used to select 704 participants. An interviewer-administered questionnaire was used to collect the data. The collected data was entered to EPI info version 7 & was analyzed by SPSS version 20. Binary logistic regression model was used to identify actual predictors of eye care service utilization. All variables were entered to multivariable analysis and variables with p-value < 0.05 were considered as statistically significant.

**Result:**

A total of 668 adults participated with a response rate of 94.9%. The median (±IQR) age of study respondents was 48(±10) years and 52.7% were females. The proportion of eye care service utilization within the past 2 years was found to be 23.8% [95% CI: 20.5%-27.1%]. Having history of eye disease [AOR = 9.8, 95% CI: 6.1–15.6], having awareness of regular eye checkup importance [AOR = 7.3, 95% CI:4.4–12.2], older age (age ≥65 years) [AOR = 5.0, 95% CI:1.9–13.3] and higher family monthly income (income ≥6000 ETB) [AOR = 3.0, 95% CI:1.5–5.7] and income 4001–5999 ETB [AOR = 2.9, 95% CI: 1.4–5.9] were positively associated with eye care service utilization.

**Conclusion and recommendation:**

The proportion of eye care service utilization among older adults in Hawassa city was low. Having a history of eye disease, having awareness about regular eye checkup importance, older age and higher family monthly income were significantly associated with utilization of eye care service. Therefore, it is recommended to provide eye health education for the community to increase awareness about eye care service utilization which can improve eye care service utilization.

## Introduction

Eye care service utilization is the use of eye care service by persons for the purpose of preventing and curing eye problems, promoting maintenance of eye health or obtaining information about one’s eye health status and prognosis [1].

Routine vision screening has been recommended by several medical organizations, including the American Academy of Ophthalmology [2, 3]. Ophthalmic and optometric best practices recommend older adults to visit an eye care professional regularly to have a comprehensive eye examination [4]. Recommended intervals between comprehensive examinations vary with age and risk factors. All individuals, particularly those with risk factors for ocular disease, should be re-examined periodically to prevent or minimize vision loss by detecting and treating the disease at an early stage. Studies have indicated that up to 40% of legal blindness could have been prevented or ameliorated if individuals had received timely ophthalmic screening and care [5].

The proportion of eye care service utilization differs widely across the world. According to previous studies; it ranges from 18%-82.5% [6–9].

The proper utilization of available eye care service is mandatory to reduce the burden of visual impairment (VI) worldwide [10]. As a part of health care service, the use of eye care service is generally influenced by a range of psychological, sociocultural, and economic factors [11]. In many parts of the developing world, eye care service is scarce and even where such service is available; the communities are not able to avail themselves [12]. Therefore this is one factor for VI to be high globally as much as, 36 million blind, 217 million moderate or severe VI and 188 million mild VI [13]. In other words, about 80% of VI is considered avoidable [14].

Ethiopia launched the Vision 2020 Initiative in September 2002 with the long-term aim of developing a sustainable comprehensive eye health care system to ensure the best possible vision for all people and thereby improve their quality of life. In spite of this, the national prevalence of blindness is 1.6% and that of low vision is 3.7% [15]. Since Ethiopia is one of the developing countries the available health care service especially eye care services are inadequate. Even where such services available the community may not utilize them. This might be due to different socio-economic factors like the patients may not afford to pay for the services. So that to reduce the burden of VI with an appropriate strategy it is essential to know the proportion of eye care service utilization and associated factors.

However, there is a scarcity of information on eye care service utilization and associated factors in Ethiopia in general and in the study area in particular. Therefore, this study was conducted to fill this gap by assessing the proportion of eye care service utilization and associated factors among older adults in Hawassa city, South Ethiopia. The result of this study may help the responsible body to increase the utilization of eye care service and thereby lessen the burden of VI in the country. Furthermore, the finding of the study will be used as baseline information for further study.

## Methods and materials

### Study design and period

A community-based cross-sectional study was conducted from April 25 to May 30, 2019.

### Study area

This study was conducted in Hawassa city, South Ethiopia. Hawassa city is located 273 km to the south of Addis Ababa, the capital city of Ethiopia. The city administration divided into 8 sub-cities and 20 urban kebeles. There are one comprehensive specialized hospital and four private eye care services providing specialty clinics which gives eye care service for the community.

### Source population

All older adults age ≥ 40 years who live permanently in Hawassa city were source of population for this study.

### Study population

All older adults age ≥ 40 years who live permanently in selected kebeles of Hawassa city were the study population.

### Sample size determination

The sample size was calculated by using a single proportion population formula. After considering the proportion of eye care service utilization 32% which was taken from a study done in Nigeria [16], 95% confidence interval, 5% marginal error, design effect of 2 and 5% non-response rate the final sample size becomes 704.

### Sampling technique

Multistage sampling technique was used to get the required sample. So that first of all 20% of total kebeles which were 4 kebeles were selected by simple random sampling. Then the sample size was proportionally allocated according to the population size of each selected kebeles. The required household was selected by systematic random sampling with an interval of 17 households (K = 17). Finally, one eligible individual was selected by simple random sampling from the selected household, if more than one eligible individual found in the selected household. If the eligible individual was not available at the time of data collection, the household was revisited two times. However, if there was no person who fulfills the inclusion criteria in the selected household at all, one eligible individual was selected from the next household.

### Data collection tool and procedures

A pre-tested structured interviewer-administered questionnaire in the Amharic language was used to collect the data. Five data collectors (BSc Optometrists) collected the data with one supervisor (MSc Optometrist). After permission was obtained from the household, data collectors explained about the study for selected study participant and get verbal informed consent from the study participant. Then they interview the participant about eye care service utilization and associated factors.

### Operational definition

#### Awareness of regular eye checkup importance

Participants who have ever heard the importance of regular eye checkup or examination were considered as they had awareness of regular eye checkup importance.

#### Eye care service utilization

If the individual reported that he/she had visited eye care service providing a center for eye checkup/examination or for eye problem at least once, within the past 2 years, it was considered as he/she utilized eye care service for this study [5].

#### Eye care service providing center

Health institution with at least one eye care service provider (ophthalmologist, optometrist, ophthalmic nurse, ophthalmic officer or cataract surgeon).

#### Has escort

Participants who had someone who will help them to visit eye care service providing centers for their eye checkup.

### Data quality assurance

The questionnaire was prepared first in English language after reviewing literatures on eye care service utilization to identify and include all factors which had association in previous studies. Then it was translated to Amharic and re-translated back to English to check for consistencies in meaning. The questionnaire was pre-tested on 35 older adults in Tukur wuha town, West Arsi zone prior to the data collection to check for completeness, appropriateness and common understanding. The items’ validity was checked by calculating Cronbach’s alpha value which was 0.8. In addition to this principal investigator gave training for data collectors and supervisor on data collection procedures, sampling technique & how to use questionnaire. There was supervision during data collection and the collected data was evaluated for its completeness daily.

### Data processing and analysis

After data cleaning and coding was done, the collected data was entered to EPI info7. Then it was exported to Statistical Package for Social Science (SPSS) version 20 for analysis. Summary statistics, frequencies, and cross-tabulations were performed for the descriptive data. Binary logistic regression model was used to identify the association between independent factors and eye care service utilization. Bivariable and multivariable logistic regression analyses were done to identify associated factors for eye care service utilization. All variables were entered with enter method to multivariable regression analysis to identify final predictors of the outcome variable. The fitness of the model was checked with Hosmer-Lemeshow model fitness test. Adjusted odds ratio with 95% confidence interval was used to measure the strength of association between predictors & the outcome variable. Those variables with p<0.05 in multivariable analysis were considered as statistically significant.

### Ethical considerations

Ethical clearance was obtained from University of Gondar College of medicine and health sciences, school of medicine ethical review committee. In addition, Official letter was obtained from University of Gondar College of medicine and health sciences, Optometry department & Hawassa city administration. Oral informed consent was obtained from each study participant after explaining the purpose of the study. To participate in the study was fully voluntary and confidentiality of the collected information from study participants was secured by coding and locking the data. Information about the importance of regular eye checkup was given for each study subject at the end of data collection. Advice was given for those with self-reported eye problem to visit eye care service providing center for eye examination and management.

## Results

### Socio-Demographic characteristics of study participants

A total of 668 adults age 40 years or above participated in this study with a response rate of 94.9%. Of the study participants, 52.7% were female. The median (±IQR) age of study respondents was 48 (±10) years. “Table 1”

**Table 1:** Socio-demographic characteristics of study participants, Hawassa city, South Ethiopia, June 2019(n = 668).

Characteristics	Frequency	Percent
**Age (in years)**		
40–54	514	76.9
55–64	104	15.6
≥ 65	50	7.5
**Sex**		
Male	316	47.3
Female	352	52.7
**Ethnicity**		
Sidama	178	26.7
Welayta	100	15.0
Amhara	129	19.3
Oromo	85	12.7
Gurage	53	7.9
Others*	123	18.4
**Religion**		
Orthodox	247	37.0
Protestant	298	44.6
Muslim	79	11.8
Catholic	23	3.5
Others**	21	3.1
**Marital status**		
Single	13	1.9
Married	569	85.2
Divorced	44	6.6
Widowed	42	6.3
**Educational level**		
Cannot write & read	58	8.7
write & read	97	14.5
Primary education	142	21.2
Secondary education	132	19.8
College and above	239	35.8
**Occupation**		
Governmental employee	160	24.0
Non-governmental employee	103	15.4
Merchant	116	17.4
Housewife	175	26.2
Retired	47	7
Others***	67	10.0
**Family monthly income in ETB**		
500–2000	202	30.3
2001–4000	189	28.3
4001–5999	97	14.5
≥ 6000	180	26.9

Others*Tigre, Silte; Others**Adventist, Wakefata; Others***Farmer, Driver, Carpenter

### Medical and medical-related characteristics of study participants

Three hundred sixty-nine (55.2%) respondents had a self-reported history of eye problem or disease and out of these one hundred sixty-three (44.4%) reported that their eye problem affects their daily activity. But only 278(41.6%) of study participants had visited eye care service providing center for an eye examination in the past. “Table 2”

**Table 2:** Medical and medical-related characteristics of study participants, Hawassa city, South Ethiopia, June 2019 (n = 668).

Characteristics	Frequency	Percent
**Health insurance**		
Yes	40	6.0
No	628	94.0
**Hypertension**		
Yes	113	16.9
No	534	79.9
I don’t know	21	3.2
**DM**		
Yes	22	3.3
No	619	92.7
I don’t know	27	4.0
**History of eye disease**		
Yes	369	55.2
No	299	44.8
**Eye problem affect daily activity (n = 369)**		
Yes	164	44.4
No	205	55.6
**Family history of eye disease**		
Yes	163	24.4
No	399	59.7
I don’t know	106	15.9
**Aware about regular eye checkup**		
Yes	399	59.7
No	269	40.3
**Ever visit eye care center for eye checkup**		
Yes	278	41.6
No	390	58.4
**Go to eye care center at a time noticed or faced eye problem (n = 278)**		
Yes	251	90.3
No	27	9.7
**Had escort**		
Yes	399	59.7
No	269	40.3

Regarding the distribution of participants who had visited eye care service providing center for eye examination of the total study participants, 37.5%, 34.8%, 30.5%, 23.8% & 13.6% had visited eye care service providing center for eye examination within the past 5 years, 4 years, 3 years, 2 years and 1 year respectively. While 1.2% of participants could not remember when they visited eye care service providing center for an eye examination.

Of those who had visited eye care service providing center for an eye examination in the past, more than half (51.8%) visit eye care service providing center for an eye examination, only if they faced eye disease or problem.

The frequently reported history of eye problems by the respondents were reduction of vision at near (28.7%), eye pain (11.4%), reduction of vision at distance (10.9%), itching (7%), redness (5.8%), tearing (3.9%) and trauma (2.4%).

### Eye care service utilization

Of the total study participants, one hundred fifty-nine (23.8%) utilized eye care service within the past 2 years (95% CI: 20.5% - 27.1%).

**Fig 1:**
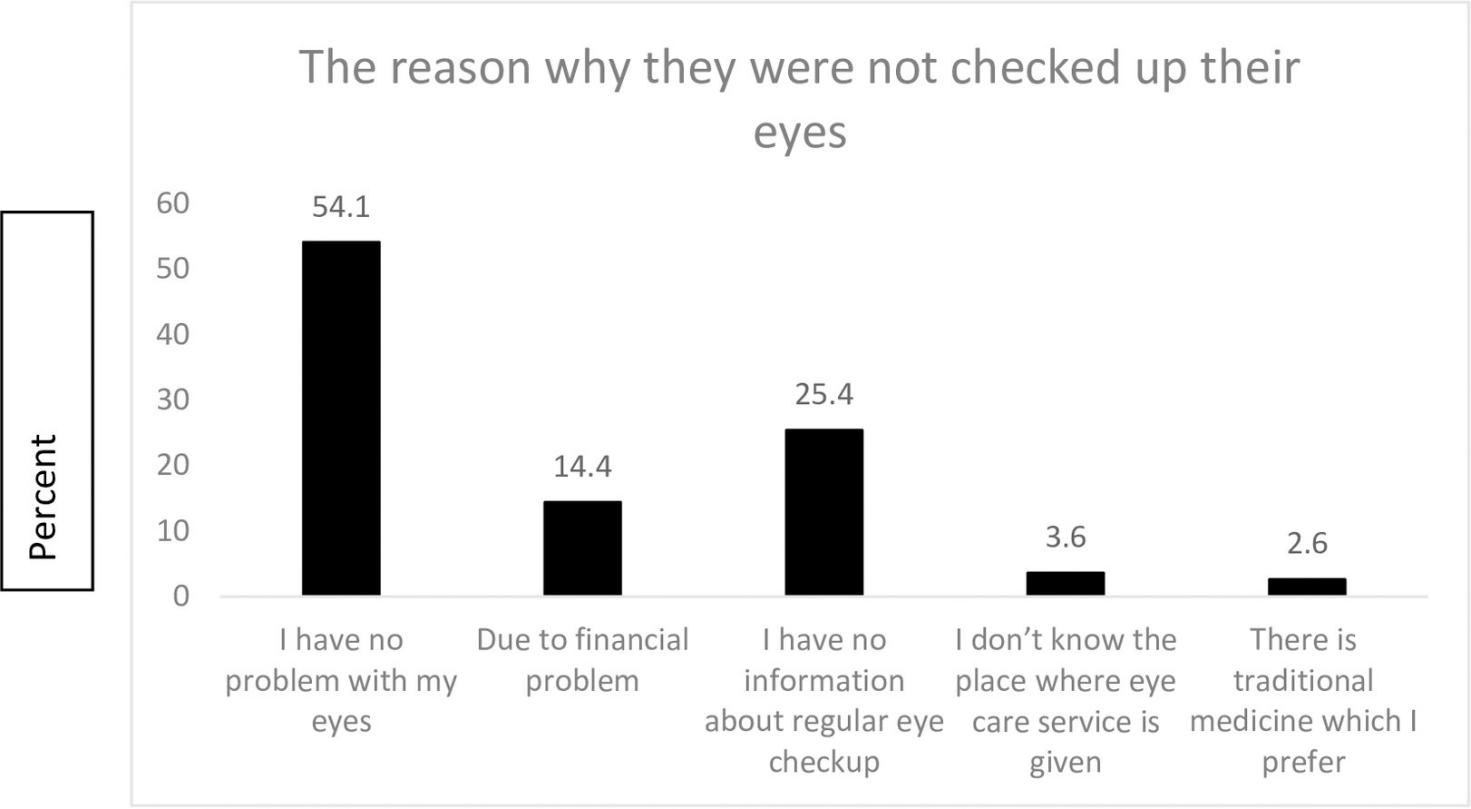
The reason for not visiting eye care service providing center for an eye examination in the past among older adults in Hawassa city, South Ethiopia, June 2019 (n = 390).

Of the total study participants, 58.4% did not visit eye care service providing center for an eye examination in the past. Out of these more than half (54.1%) reported that they were not visited eye care service providing center for eye examination because they feel that they had no problem with their eye. “Fig 1”

The preferred place of choice by most respondents for eye examination when they faced eye problems or for regular eye checkup were eye care center (45.4%). “Table 3”

**Table 3:** The preferred place of choice by respondents for eye examination when they faced eye problem or for a regular eye checkup, Hawassa city, South Ethiopia, June 2019 (n = 668).

Place of choice	Frequency	Percent
Eye care center	303	45.4
General Hospital	208	31.1
Health center	88	13.2
Traditional medicine	16	2.4
Holy water	7	1
Nowhere	46	6.9

### Previous history of eye disease of the study participants

Among study participants, 369(55.2%) of them had a self-reported history of eye disease. Of participants who had history of eye disease, 232(62.9%) participants utilize eye care services needing curative care but 46(12.5%) participants who didn’t have a history of eye problems visit eye care service providing centers for preventive care. “Table 4”

**Table 4:** Previous history of eye disease of study participants, Hawassa city, South Ethiopia, June 2019 (n = 668).

Characteristics	History of eye disease	
	No (n)	Yes (n)
**Age (in years)**		
40–54	264	250
55–64	27	77
≥ 65	8	42
**Sex**		
Male	120	196
Female	179	173
**Ethnicity**		
Sidama	79	99
Welayta	46	54
Amhara	51	78
Oromo	39	46
Gurage	28	25
Others*	56	67
**Religion**		
Orthodox	100	147
Protestant	135	163
Muslim	45	34
Catholic	13	10
Others**	6	15
**Marital status**		
Single	6	7
Married	268	301
Divorced	15	29
Widowed	10	32
**Educational level**		
Cannot write & read	31	27
Can write & read	43	54
Primary education	86	56
Secondary education	62	70
College and above	77	162
**Occupation**		
Governmental employee	58	102
Non-governmental employee	46	57
Merchant	65	51
Housewife	92	83
Retired	10	37
Others***	28	39
**Family monthly income in ETB**		
500–2000	96	106
2001–4000	98	91
4001–5999	45	52
≥ 6000	60	120
**Health insurance**		
Yes	19	21
No	280	348
**Hypertension**		
Yes	37	76
No	249	285
I don’t know	13	8
**DM**		
Yes	5	17
No	280	339
I don’t know	14	13
**Eye problem affect daily activity (n = 369)**		
Yes	1	163
No	1	204
**Family history of eye disease**		
Yes	68	95
No	172	227
I don’t know	59	47
**Aware about regular eye checkup**		
Yes	150	249
No	149	120
**Utilize eye care service**		
Yes	46	232
No	253	137
**Had escort**		
Yes	192	207
No	107	162

### Factors associated with eye care service utilization

In multivariable logistic regression analysis, having a history of eye disease/problem, having awareness of regular eye checkup importance, older age and higher family monthly income were significantly associated with utilization of eye care service at 0.05 level of significance. “Table 5”

**Table 5:** Factors associated with eye care service utilization among older adults in Hawassa city, South Ethiopia, June 2019 (n = 668).

	Utilization(N)			
Variable	Utilized	Not utilized	COR(95% CI)	AOR(95% CI)
Age(in years)				
40–54	107	407	1.0	1.0
55–64	30	74	1.5(1.0–2.5)	3.4(1.8–6.4) ***
≥ 65	22	28	3.0(1.6–5.4)***	5.0(1.9–13.3) ***
Sex				
Male	86	230	1.4(1.0–2.0)	1.2(0.7–2.0)
Female	73	279	1.0	1.0
Ethnicity				
Sidama	43	135	1.0	1.0
Welayta	22	78	0.9(0.5–1.6)	0.5(0.3–1.1)
Amhara	29	100	0.9(0.5–1.6)	0.8(0.4–1.6)
Oromo	27	58	1.5(0.8–2.6)	1.2(0.6–2.6)
Gurage	11	42	0.8(0.4–1.7)	1.23(0.5–2.9)
Others*	27	96	0.9(0.5–1.5)	0.8(0.4–1.5)
Marital status				
Married	136	433	1.0	1.0
Single	5	8	2.0(0.6–6.2)	0.6(0.1–2.7)
Divorced	8	36	0.7(0.3–1.6)	1.0(0.4–2.5)
Widowed	10	32	1.0(0.5–2.1)	2.8(1.1–7.2)
Educational level				
Cannot write & read	7	51	1.0	1.0
Can write & read	16	81	1.4(0.6–3.7)	0.6(0.2–1.7)
Primary education	15	127	0.9(0.3–2.2)	0.9(0.3–2.3)
Secondary education	29	103	2.1(0.8–5.0)	0.7(0.3–1.8)
College and above	92	147	4.6(2.0–10.5)***	1.5(0.5–4.2)
Occupation				
House wife	26	149	1.0	1.0
Governmental employee	55	105	3.0(1.8–5.1)***	0.8(0.4–1.9)
Non-governmental employee	29	74	2.3(1.2–4.1)**	0.9(0.4–2.2)
Merchant	20	96	1.2(0.6–2.3)	0.7(0.3–1.6)
Retired	17	30	3.3(1.6–6.7)**	1.1(0.4–3.2)
Others***	12	55	1.3(0.6–2.7)	1.9(0.2–2.4)
Family monthly income in ETB				
500–2000	28	174	1.0	1.00
2001–4000	31	158	1.2(0.7–2.1)	1.7(0.9–3.2)
4001–5999	24	73	2.0(1.1–3.8)*	2.9(1.4–5.9) ***
≥ 6000	76	104	4.5(2.8–7.5)***	3.0(1.5–5.7) ***
Health insurance				
Yes	10	30	1.1(0.5–2.2)	1.2(0.5–3.0)
No	149	479	1.0	1.0
Hypertension				
No	120	414	1.0	1.0
Yes	34	79	1.5(0.9–2.3)	0.9(0.5–1.7)
I don’t know	5	16	1.1(0.4–3.0)	2.3(0.7–7.8)
DM				
No	138	481	1.0	1.0
Yes	12	10	4.2(1.8–9.9)**	0.7(0.2–2.4)
I don’t know	9	18	1.7(0.8–4.0)	1.2(0.4–3.5)
History of eye disease				
Yes	129	240	4.8(3.1–7.4)***	9.8(6.1–15.6) ***
No	30	269	1.0	1.0
Family history of eye disease				
No	104	295	1.0	1.0
Yes	40	123	0.9(0.6–1.4)	0.8(0.5–1.4)
I don’t know	15	91	0.5(0.3–0.8)*	0.8(0.4–1.5)
Aware about regular eye checkup				
Yes	142	257	8.2(4.8–13.9)***	7.3(4.4–12.2) ***
No	17	252	1.0	1.0
Had escort				
Yes	103	296	1.3(0.9–1.9)	1.4(0.9–2.3)
No	56	213	1.0	1.0

P <0.05*, P< 0.01**, P<0.001***

Participants who had awareness about regular eye checkup importance were 7.3 times more likely to utilize eye care service [AOR = 7.3, 95% CI: 4.4–12.2] as compared to participants who didn’t have awareness about regular eye checkup importance. Similarly, participants who had a history of eye disease/problem were 9.8 times more likely to utilize eye care service [AOR = 9.8, 95% CI: 6.1–15.2] as compared to participants who had no history of eye disease/problem.

Regarding age, individuals in the age group ≥ 65 years were 5.0 times more likely to utilize eye care service [AOR = 5.0, 95% CI: 1.9–13.3] as compared to individuals in the age group 40–54 years.

Furthermore, participants with family monthly income of ≥ 6000 ETB were 3 times more likely to utilize eye care service [AOR = 3.0, 95% CI: 1.5–5.7] and participants with family monthly income of 4001–5999 ETB were 2.9 times more likely to utilize eye care service [AOR = 2.9, 95% CI: 1.4–5.9] as compared to participants with family monthly income of 500–2000 ETB.

The forward stepwise logistic regression was done by entering predisposing factors (age, sex, educational status, family history of eye disease, marital status, ethnicity and awareness about regular eye checkup) first. Then enabling factors (occupation, income, health insurance and having escort) and finally needing factors (history of DM, history of HTN and history of eye disease) were entered to the model. The result of the first model of forward stepwise logistic regression analysis showed that having higher educational status, older age and having awareness about regular eye checkup have significant association with eye care service utilization. After adjusting for predisposing factors high family monthly income was the only enabling factor which had significant association with eye care service utilization. In addition, age and awareness about regular eye checkup had significant association with eye care service utilization but the association between eye care service utilization and educational status becomes non-significant. In the final model after adjusting for predisposing and enabling factors having history of eye disease[AOR = 8.4, 95% CI (5.4–13.0)], older age[AOR = 6.4, 95% CI (2.9–14.1)], having awareness about regular eye checkup[AOR = 6.9, 95% CI (4.3–11.3)] and having high family monthly income [AOR = 2.9, 95% CI (1.6–5.3)] had significant association with eye care service utilization. “Table 6”

**Table 6:** Forward stepwise logistic regression result of eye care service utilization among older adults in Hawassa city, South Ethiopia, June 2019.

	Variables	Eye care service utilization (AOR (95%CI))
Model I	Educational status	
	Unable to read and write	1.0
	Can read and write	0.8(0.4–1.8)
	Primary education	0.8(0.4–1.9)
	Secondary education	0.9(0.4–2.0)
	College and above	2.3(1.1–5.0)*
	Age in years	
	40–54	1.0
	55–64	3.9(2.4–6.6)*
	≥65	8.4(4.0–17.7)*
	Awareness about regular eye checkup	
	No	1.0
	Yes	5.9(3.8–9.1)*
Model II	Educational status	
	Unable to read and write	1.0
	Can read and write	0.7(0.3–1.7)
	Primary education	0.8(0.3–1.7)
	Secondary education	0.7(0.3–1.6)
	College and above	1.5(0.7–3.3)
	Age in years	
	40–54	1.0
	55–64	4.1(2.4–7.0)*
	≥65	10.8(5.1–23.1)*
	Family monthly income in ETB	
	500–2000	1.0
	2001–4000	1.5(0.9–2.5)
	4001–5999	2.3(1.3–4.3)*
	≥6000	2.9(1.7–5.0)*
	Awareness about regular eye checkup	
	No	1
	Yes	5.8(3.8–9.1)*
Model III	Educational status	
	Unable to read and write	1.0
	Can read and write	0.6(0.2–1.5)
	Primary education	0.7(0.3–1.7)
	Secondary education	0.6(0.2–1.4)
	College and above	1.1(0.4–2.6)
	Age in years	
	40–54	1.0
	55–64	3.3(1.9–5.8)*
	≥65	6.4(2.9–14.1)*
	Awareness about regular eye checkup	
	No	1.0
	Yes	6.9(4.3–11.3)*
	Family monthly income in ETB	
	500–2000	1.0
	2001–4000	1.7(0.9–3.0)
	4001–5999	2.7(1.4–5.4)*
	≥6000	2.9(1.6–5.3)*
	History of eye disease	
	No	1.0
	Yes	8.4(5.4–13.0)*

## Discussion

In this study, the proportion of eye care service utilization within the past 2 years was found to be 23.8% [95% CI: 20.5% - 27.1%]. This result is higher than findings from a study done in Southwestern Nigeria (19%) [8] and World-wide basis (18%) [6]. This might be due to differences in socio-economic status of the study population, sample size and study setting. The current study was conducted among urban residents while the study in southwestern Nigeria was conducted among the rural community. Urban residents have better awareness about health, more access to health care facility and got health information from different Media as compared to rural residents. While the study conducted worldwide basis was conducted among a large sample size in different countries. Therefore, these factors may have a contribution to a higher proportion of eye care service utilization in the current study.

The current study finding is lower than the report from study conducted in Ghana (32.2%) [17], Edo state of Nigeria (32%) [16], Abuja, Nigeria (38%) [18], Limpopo province of South Africa (62.7%) [10], Pakistan (45.3%) [19] and South Korea (73.5%) [2]. This discrepancy might be due to the difference in the operational definition used for eye care service utilization in the current study and in previous studies. In the present study, all participants who reported visiting eye care service providing center for an eye examination at least once within the past 2 years were considered as they utilized eye care service. While visiting eye care service providing center for an eye examination at least once in the past or in the lifetime was considered as utilizing eye care service in previous studies. In other words, the findings from previous studies were cumulative proportion. It is obvious lifetime utilization is higher than utilization within the past 2 years.

The proportion of eye care service utilization in the current study (23.8%) is lower than reports from studies conducted in; KwaZulu-Natal province of South Africa (29.4%) [20], Canada (57%) [21], America (36%) [22], & Australia (67%-82.5%) [9]. This might due to less availability & affordability of eye health facility in the present setting, the difference in the age range of study subjects; in the current study ≥ 40 years while in most of the previous studies ≥ 50/60 years. Since most of the degenerative eye diseases occur among the older age group, this enforces them to utilize eye care service more than younger age group. In addition, the population in current study might have lower awareness about regular eye checkup and poor socio-economic status as compared to others. These might be the reason why proportion of eye care service utilization in current study is lower as compared to others.

In this study having awareness about regular eye checkup importance was positively associated with eye care service utilization. This is in consistence with a report from a study done in South Africa [23]. This is because community awareness about eye health and periodic eye checkup is the key component that enables the community to utilize available eye care service.

According to the result from the present study, participants who had a history of eye disease/problem were more likely to utilize eye care service as compared to participants who had no history of eye disease/problem. Similar findings reported from a study conducted in Australia [9], South Korea [2], Pakistan [19] & USA [24]. Most of the time patient visit eye care centers for the felt need or to relief from eye problems. If an individual disturbed by an eye problem, he/she is enforced to visit eye care center to overcome his/her problem. Therefore, people who have eye disease/problem seek eye care service more than people who have no eye disease/problem.

Older age was positively associated with utilization of eye care service in the present study. This is in harmony with findings from a study conducted in Abuja, Nigeria [18], South Korea [2], Tehran [25], Australia [9] and America [22]. Most of the major eye diseases/problem such as presbyopia, glaucoma, age-related cataract, diabetic retinopathy, age-related macular degeneration are age-related which progress with time [5]. Therefore, eye diseases affect an individual more and more as age increase. This enforces the older age group to seek eye care more than the younger age group.

This study also revealed a positive association between higher family monthly income and eye care service utilization. This is in agreement with the results reported from the study conducted in USA [24, 26], Canada [21], and World-wide basis [6]. Economic status is one key factor that determines the utilization of eye care service. An individual with higher income can afford medical payments and so utilize health service more than individuals with lower income.

Even though this study was a community-based study with adequate sample size and good response rate, it has some limitations such as the data were self-reports of study participants, so that its accuracy may be affected by recall bias or social desirability bias. Since it is a cross-sectional study, it cannot show the temporal relationship of predictors and outcome variable. The variables history of eye disease and frequently reported eye problems were based on participants reported symptoms which don’t give adequate information about specific eye condition of each participant.

## Conclusion

The proportion of eye care service utilization among older adults in Hawassa city was low. Most of the respondents who had utilized eye care service reported that they visit eye care service only if they faced eye problem or noticed eye disease. In another way, the reason why most participants did not utilize eye care service was because they thought that they have no problem with their eye. This shows only a low percentage of study participants visit eye care center for regular eye checkup as recommended. Having a history of eye disease/problem, having awareness about the need for regular eye checkup, older age and higher family monthly income were significantly associated with more utilization of eye care service among older adults in Hawassa city. This implies providing eye health education for the community is paramount in improving eye care service utilization.

## Supporting information

S1 Data (DOCX)
