# Supplementary material for: Eye care service utilization and associated factors among older adults in Hawassa city, South Ethiopia
Source: PLoS One. 2020 Apr 16;15(4):e0231616. doi: 10.1371/journal.pone.0231616 (PMC7162464; doi:10.1371/journal.pone.0231616)
Supplement: S1 Data — (DOCX) [file pone.0231616.s001.docx]

**Operational definition**

**Awareness of regular eye checkup importance:** Participants who have you ever heard the importance of regular eye checkup or examination were considered as they had awareness of regular eye checkup importance.

**Eye care service utilization:** if the individual reported that he/she had visited eye care service providing a center for eye checkup/examination or for eye problem at least once, within the past 2 years, it was considered as he/she utilized eye care service for this study ([5](#_ENREF_5)).

**Eye care service providing center:** Health institution with at least one eye care service provider (ophthalmologist, optometrist, ophthalmic nurse, ophthalmic officer or cataract surgeon).

ANNEX 1: ENGLISH VERSION QUESTIONNAIRE

ID_____________

| **Part 1. Socio-demographic characteristic** | | |
| --- | --- | --- |
| Serial NO | Question | Response |
| 1 | Age | _________________ |
| 2 | Sex | 1. Male 2. Female |
| 3 | Ethnicity | 1. Sidama 2. Welayta 3. Ahmara 2. Oromo 5. Gurage 6. Others |
| 4 | Religion | 1. Orthodox 2.Protestant 3. Muslim  4. Catholic 5. Others |
| 5 | Marital Status | 1. Single 2.Married  3.Divorced 4.Widowed |
| 6 | Educational level | 1. cannot write and read  2.can write and read only  3.Primary Education  4. Secondary Education  5. College and Above |
| 7 | Occupation | 1.Govermental Employee  2.Non-Governmental Employee  3.Marchent  4.House wife  5. Retired  6. Farmer 7. Others |
| 8 | Average family monthly income in ETB | --------------------------- |

|  | **Part 2 Medical and eye care service utilization related question** | |
| --- | --- | --- |
| Serial  NO | Question | Response |
| 9 | Do you have health insurance? | 1. Yes 2. No |
| 10 | Do you have hypertension? | 1. Yes 2. No 3. I don’t know |
| 11 | Do you have diabetes mellitus? | 1. Yes 2. No 3. I don’t know |
| 12 | Did you have history of eye disease/problem? | 1. Yes  2. No , **If the answer is no go to question #15** |
| 13 | Which type of eye problem do you had? | 1. Reduction of vision at distance  2. Reduction of vision at near  3. Pain 4. Redness  5. Itching 6. Tearing  7. Eye trauma 8. specify if other -------------- |
| 14 | Does your eye problem affect your daily activity? | 1. Yes 2. No |
| 15 | Is there anyone who has history of eye disease from your family? | 1. Yes 2. No 3. I don’t know |
| 16 | Have you ever heard the importance of regular eye checkup or examination? | 1. Yes 2. No |
| 17 | Have you ever heard when an individual should go to eye care service providing center for his/her eye examination or checkup? | 1. Yes 2. No |
| 18 | Did you ever visit eye care service providing center for your eye checkup? | 1.Yes  2. No, **If the answer is no go to question #22** |
| 19 | When did you visit eye care service providing center for your eye examination recently? | 1. Within last 1year 6. Before 5 years  2. Within last 1-2 year 7. I can’t remember  3. Within last 2-3 year  4. Within last 3-4 year  5. Within last 4-5 year |
| 20 | How often do you visit eye care service providing center for your eye checkup? | 1.Once a year or more  2.Once within two years  3.Once within three years  4.Once within four years  5.I go to eye clinic only if I had eye disease |
| 21 | Did you go to eye care service providing center for your eye examination at a time you noticed having eye disease or face eye problem? | 1. Yes **go to question #23**  2. No |
| 22 | Why you didn’t have been examined or checked up your eye at eye care center? | 1.Because I have no problem with my eye  2.Due to financial problem  3.I don’t have information about importance of eye checkup  4.I don’t know where the eye care service is given  5.There is a traditional medicine which I prefer |
| 23 | Do you have an escort who help you to visit care center for eye checkup? | 1.Yes  2.No |
| 24 | Where do you go for your eye examination or checkup? | 1.Eye care center  2.General hospital  3.Health center  4.Holy water  5.Traditional medicine  6. No where |

ANNEX 2: AMHARIC VERSION QUESTIONNARIE

               ኮድ _____________

| ክፍል 1. ማህበራዊ መረጃዎች | | |
| --- | --- | --- |
| ተ. ቁ | ጥያቄ | መልስ |
| 1 | ዕድሜ | -------------------- |
| 2 | ፆታ | 1 ወንድ 2. ሴት |
| 3 | ብሄር | 1. ሲዳማ 2. ወላይታ 3. አማራ 4. ኦሮሞ 5. ጉራጌ 6. ሌላ |
| 4 | ሃይማኖት | 1. ኦርቶዶክስ 2. ፕሮቴስታነት 3. ሙስሊም  4. ካቶሊክ 5. ሌላ |
| 5 | የጋብቻ ሁኔታ | 1. ያላገባ/ች 2. ያገባ/ች  3. የፈታ/ች 4. የሞተበት/ባት |
| 6 | የትምህርት ደረጃ | 1. ማንበብና መፃፍ የማይችል  2. ማንበብና መጻፍ ብቻ የሚችል  3. አንደኛ ደረጃ  4. ሁለተኛ ደረጃ  5.ኮሌጅ/ ከዚያ በላይ |
| 7 | ሥራ | 1. የመንግስት ሰራተኛ  2. መንግስታዊ ያልሆነ ተቋም ሰራተኛ  3. ነጋዴ 4. የቤት እመቤት  5.ጡረታ 6. ገበሬ  7. ሌላ |
| 8 | የቤተሰብ ወርሃዊ ገቢ በአማካይ በኢትዮጵያ ብር | --------------------------- |

|  | ክፍል 2 የሕክምና እና የዓይን ሕክምና አገልግሎት አጠቃቀም ጋር የተያያዘ ጥያቄ | |
| --- | --- | --- |
| ተ. ቁ | ጥያቄ | መልስ |
| 9 | የጤና መድን ሽፋን አለዎት? | 1. አዎ 2. የለኝም |
| 10 | የደም ግፊት አለብዎት? | 1. አዎ 2. የለብኝም 3. አላውቅም |
| 11 | የስኳር በሽታ አለብዎት? | 1. አዎ 2. የለብኝም 3. አላውቅም |
| 12 | ዓይኖትን ታሞ ያውቃሉ ? | 1. አዎ  2. አለዉቅም፣**መልስ አለዉቅም ከሆነ ወደ ጥያቄ #15 ሂዱ** |
| 13 | የትኛው የዓይን ሕመም ነዉ  የታመሙት ? | 1. የርቀት እይታ መቀነስ 2. የቅርብ እይታ መቀነስ  3. የኣይን ህመም 4. የኣይን መቅለት  5. ማሳከክ 6. ማንባት  7. የኣይን ምት 8. ሌላ ካለ ይግለጹ. ------- |
| 14 | የዓይን ችግርዎ በዕለት ተዕለት እንቅስቃሴዎ ላይ ተጽዕኖ ያሳድራል? | 1. አዎ 2. አይደለም |
| 15 | ከቤተሰብዎ ውስጥ የዓይን ሕመም ያለበት ሰው አለ ? | 1. አዎ 2. የለም 3. አላውቅም |
| 16 | በየጊዜው የኣይን ምርመራ ማድረግ አስፈላጊ እንደሆነ ሰምተው ያውቃሉ? | 1. አዎ 2. ሰምቼ አለዉቅም |
| 17 | አንድ ሰዉ ለዓይን ምርመራ ወደ ኣይን ሕክምና ማዕከል መቼ መሄድ እንዳለብት ሰምተው ያውቃሉ? | 1. አዎ 2. አለዉቅም |
| 18 | ለዓይን ምርመራ ወደ ዓይን ሕክምና ማዕከል ሄደው ያውቃሉ? | 1. አዎ  2. አለዉቅም፣ **መልስ አለዉቅም ከሆነ ወደ ጥያቄ**  **#22 ሂዱ** |
| 19 | ለአይን ምርመራ ወደ ኣይን ሕክምና ማዕከል በቅርብ የሄዱት መቼ ነው? | 1. በለፈዉ 1 ዓመት ውስጥ 5. በለፈዉ 4-5 ዓመት ውስጥ  2.በለፈዉ 1-2 ዓመት ውስጥ 6. ከኣምስት ዓመት በፊት  3. በለፈዉ 2-3 ዓመት ውስጥ 7. አላስታውስም  4. በለፈዉ 3-4 ዓመት ውስጥ |
| 20 | ለአይን ምርመራ በያ ስንት ግዜ ወደ የኣይን ሕክምና ማዕከል ይሄዳሉ? | 1. በዓመት ኣንዴ ወይም ከዚያ በላይ 2. በሁለት ዓመት ውስጥ ኣንዴ 3. በሶስት ዓመት ውስጥ ኣንዴ 4. በአራት ዓመታት ውስጥ ኣንዴ 5. የዓይን ሕመም ወይም ችግር ስኖርኝ ብቻ ወደ የኣይን ሕክምና ማዕከል እሔዳለሁ |
| 21 | የዓይን በሽታ ወይም ችግር እንደአለቦት ባወቁ ጊዜ ወደዉኑ ወደ ዓይን ሕክምና ማዕከል ለአይን ምርመራ ይሄዳሉ? | 1. አዎ **ወደ ጥያቄ**  **#23 ሂዱ**  2. አለሄድም |
| 22 | እስከ ኣሁን ለምን አይኖትን ሳይመረመሩ ቆዩ? | 1. በዓይኔ ምንም ችግር የለብኝም 2. የሕክምና ገንዘብ ስላለለኝ 3. ስለዓይን ምርመራ አስፈላጊነት መረጃ የለኝም 4. የአይን ሕክምና አገልግሎት የት እንደሚሰጥ አላውቅም 5. የምመርጥ የባህላዊ መድሃኒት አለ |
| 23 | ለአይን ምርመራ የኣይን ሕክምና ማዕከል እንዲጎበኙ የሚያግዝ ሰው አለዎት? | 1. አዎ  2. የለኝም |
| 24 | ለዓይን ምርመራዎ ወይም ሕክምና የት ነው የሚሄዱት? | 1. የኣይን ሕክምና ማዕከል 2. ጠቅላላ ሆስፒታል 3. ጤና ጣቢያ 4. ፀባል 5. የባላዊ ህክምና 6. የትም ቦታ አልሄድም |
